# Intestinal Colonization by a *Lachnospiraceae* Bacterium Contributes to the Development of Diabetes in Obese Mice

**DOI:** 10.1264/jsme2.ME14054

**Published:** 2014-10-04

**Authors:** Keishi Kameyama, Kikuji Itoh

**Affiliations:** 1 Institute for Innovation, Ajinomoto Co., Inc., Kawasaki 210–8681, Japan; 2 Department of Veterinary Public Health, Graduate School of Agricultural and Life Sciences, The University of Tokyo, Tokyo 113–8657, Japan

**Keywords:** gut microbiota, diabetes, T-RFLP, gnotobiotic mouse, *Lachnospiraceae*

## Abstract

The aim of the present study was to identify bacteria that may contribute to the onset of metabolic dysfunctions. We isolated and identified a candidate bacterium belonging to *Lachnospiraceae* (strain AJ110941) in the feces of hyperglycemic obese mice. The colonization of germ-free *ob/ob* mice by AJ110941 induced significant increases in fasting blood glucose levels as well as liver and mesenteric adipose tissue weights, and decreases in plasma insulin levels and HOMA-β values. These results indicated that the specific gut commensal bacterium AJ110941 influenced the development of obesity and diabetes in *ob/ob* mice with genetic susceptibility for obesity.

The gut microbiota has been shown to influence host energy homeostasis, metabolism, and inflammation; thus, microbial communities within the gut are now recognized as an important environmental factor in the onset of obesity and type 2 diabetes (T2D) (5, 7, 12, 14, 23). As a consequence, interest in the development of methods to target gut microbiota as a therapy for T2D is increasing (2, 12, 18, 21).

Several studies have conducted metagenomic analyses in an attempt to identify members of the gut microbial community that contribute most significantly to the progression of T2D. Qin *et al.* performed a metagenomic analysis on fecal samples obtained from 345 Chinese men and women with or without T2D (19). Karlsson *et al.* also conducted a metagenomic analysis to compare microbial communities in fecal samples collected from 145 European women who had either T2D, impaired glucose metabolism, or were healthy (13). Both of these studies reported that butyrate-producing *Roseburia* species and *Faecalibacterium prauznitzii* were rarer in subjects with T2D; however, the identified bacteria that existed in high population in T2D subjects differed between these studies (8). Cani *et al.* proposed that lipopolysaccharide (LPS), a cell wall component of almost all Gram-negative bacteria, may be a key substance responsible for metabolic endotoxemia, low-grade systemic inflammation, and insulin resistance in mouse models (1, 3). In support of this, a previous study reported that experimental endotoxemia induced adipose inflammation and insulin resistance in human subjects (17).

We herein searched for the specific gut commensal bacterium related to metabolic syndrome using the terminal restriction fragment length polymorphism (T-RFLP) analysis of fecal samples from a mouse model of T2D. In this study, a comparison of homozygous *db/db* (diabetic) with heterozygous *db/*+ (non-diabetic) mice revealed that a specific fragment of the T-RFLP analysis was present at significantly higher levels in *db/db* mice than in *db/*+ mice. Even though *ob/ob* obese mice gain weight rapidly and show the symptoms of insulin resistance, most of the obese mice do not develop severe hyperglycemia compared with *db/db* mice (22). However, one of the *ob/ob* mice in the present study showed an abnormally high fasting blood glucose (FBG) level similar to *db/db* mice. The specific fragment observed in *db/db* mice was also the most prominent in the abnormally hyperglycemic *ob/ob* mouse. We hypothesized that the specific bacterium harboring the fragment may have contributed to the progression of T2D; therefore, we isolated and cultured the bacterium (strain AJ110941). We then determined that colonization by the isolate with the LPS producer *E. coli* induced hyperglycemia and the accumulation of adipose tissue in the gnotobiotic *ob/ob* mouse model.

All experimental procedures were reviewed and approved by the Animal Care Committee of Ajinomoto Co., Inc. Male 5-week-old homozygous BKS.Cg-Dock7^m^ +/+Lepr^db^/J (*db/db*, diabetic) mice, heterozygous control (*db/*+, non-diabetic) mice, and homozygous B6.V-Lep^ob^/J (*ob/ob*, obese) mice were obtained from Charles River Japan (Yokohama, Japan). Male 5-week-old germ-free *ob/ob* mice were obtained from Sankyo Lab Service (Tokyo, Japan). These mice were housed in a controlled environment (on a 12-h light/dark cycle with lights turning off at 19:00) with free access to standard chow CRF-1 (Oriental Yeast, Tokyo, Japan) and water and kept in specific pathogen-free (SPF) or germfree/gnotobiotic conditions throughout the experimental period. Fresh feces and blood were collected from 5- and 11-week-old mice after fasting for 16 h.

Blood glucose (FBG) levels were determined using DRI-CHEM 7000V (Fujifilm, Tokyo, Japan). Plasma insulin levels were determined using an ultrasensitive mouse insulin kit (Morinaga Institute of Biological Science, Yokohama, Japan). Plasma glucagon levels were determined using the Glucagon ELISA Kit Wako (Wako Pure Chemical Industries, Osaka, Japan). Insulin resistance (HOMA-IR) and β-cell function (HOMA-β) were both calculated on the basis of the fasting levels of plasma glucose and insulin according to the homeostasis model assessment (HOMA) method (16).

Fresh fecal samples were collected from mice, added to 99% ethanol, and stored at −30°C. Bacterial DNA was extracted from the fecal samples using the FastDNA spin kit for soil (MP Biomedicals, Santa Ana, CA) using the FastPrep instrument (MP Biomedicals). T-RFLP analyses of the mouse gut microbiota were performed as previously described (10). Two universal primers, 27F labeled with 6-carboxyfluorescein (FAM): 5′-FAM-AGAGTTTGATCCTGGCTCAG-3′ and 1492R: 5′-GGTTACCTTGTTACGACTT-3′ were used in PCR to amplify the 16S rRNA gene coding region. Purified PCR products of the 16S rRNA gene were digested with *Msp*I (Takara Bio, Otsu, Japan).

To isolate and culture the bacterium, fresh feces were collected and immediately weighed and transferred to an anaerobic chamber. The feces were homogenized with a 50-fold volume (v/w) of an anaerobic diluent, serially diluted, and then plated on Eggerth-Gagnon (EG) agar plates (11). The agar plates were incubated at 37°C for 4 d in the anaerobic chamber. All of the colonies were identified by their fragment size, and a target colony with a fragment size of 282 bp was then selected. The colony was passaged onto new EG agar plates, and the bacterial purity of the isolate was ensured by re-streaking and microscopic observations. The 16S rRNA gene sequence of the isolated bacterium was determined as previously described (15). The sequence was assembled using GENETYX version 7 (GENETYX, Tokyo, Japan). The identification and phylogenic tree analysis of the 16S rRNA gene sequence were carried out using the Ribosomal Database Project (RDP) (6).

When the mice were 8 weeks old, germ-free *ob/ob* mice were separated into 3 groups for the germ-free/gnotobiotic experiment: Group-1 (*n*=4), germ-free; Group-2 (*n*=4), colonization by the non-pathogenic *Escherichia coli* strain E-17, which had been isolated from SPF mice; Group-3 (*n*=4), colonization by AJ110941 with *E. coli* E-17. Groups-2 and -3 were then orally inoculated with *E. coli* E-17 or AJ110941 with *E. coli* E-17 (approximately 1×10^8^ cells suspended in anaerobic PBS, respectively). Group-1 was orally administered PBS only. These mice were maintained under the germ-free or gnotobiotic conditions for 8 weeks.

We compared differences in gut microbiota patterns between *db/*+ and *db/db* mice. Hyperglycemia was observed in 11-week-old, but not in 5-week-old mice (Fig. S1). The 282 bp fragment was significantly higher in *db/db* than *db/*+ mice at both 5 and 11 weeks old (Fig. S2). We then evaluated the presence of the 282 bp fragment in another metabolic syndrome model, the *ob/ob* mouse. The highest FBG level observed among all *ob/ob* mice was in Mouse Number 4 (Fig. S3). The 282 bp fragment was also more prominent in hyperglycemic Mouse Number 4 than in the normal glycemic *ob/ob* mouse at both 5 and 11 weeks old (Fig. S4). Based on these *db/db* and *ob/ob* mice results, we focused on this 282 bp fragment bacteria.

We isolated a colony harboring the 282 bp fragment and established an axenic strain (strain AJ110941). The isolate was determined to be closely related to the genus *Anaerostipes* in the family *Lachnospiraceae* (Fig. 1).

We then generated gnotobiotic *ob/ob* mice colonized by AJ110941 with *E. coli*. Eight weeks after the inoculation, the cecum, liver, adipose tissue, and blood were collected after a 16-h fast. The gut microbiota of the cecal contents from the three groups was determined by T-RFLP analysis. No PCR amplicon was detected in Group-1. Only a single fragment (488 bp) that corresponded to *E. coli* was detected in Group-2. Only two fragments (282 bp and 488 bp) were detected in Group-3. These results clearly indicated that germ-free or gnotobiotic conditions were maintained during the experimental period. The weights of the liver and mesenteric adipose tissue significantly increased in Group-3, whereas no significant difference was observed in body weights between the three groups. FBG and plasma glucagon levels were significantly higher in Group-3, while plasma insulin levels were significantly lower. On the other hand, no significant differences were observed in these parameters between Groups-1 and -2. The homeostasis model assessment was calculated from FBG and plasma insulin levels as an index of insulin resistance (HOMA-IR) and pancreatic β cell function (HOMA-β). HOMA-β was significantly lower in Group-3, whereas no significant differences were noted in HOMA-IR between the three groups (Table 1). These results suggested that colonization by AJ110941 may have promoted the dysfunction of pancreatic β-cells.

Therefore, AJ110941 should be regarded as one of the important causative gut bacteria for the induction of T2D. A previous study reported that the relative abundance of the taxonomic family *Lachnospiraceae* was increased by early-life subtherapeutic antibiotic treatments in an obese mouse model (4). Additionally, a metagenomic study indicated that the taxonomic family *Lachnospiraceae* may be associated with T2D (19). However, it remains unclear whether bacteria belonging to the family *Lachnospiraceae* actually affect obesity and FBG levels *in vivo*. Therefore, we generated gnotobiotic *ob/ob* mice colonized by AJ110941 with *E. coli* to reveal a possible causal relationship. In a preliminary examination, we microscopically observed that AJ110941 did not singly colonize the intestinal tract of *ob/ob* germ-free mice. *E. coli*, which is a facultative anaerobe, may be needed to maintain an oxygen-free environment in the intestinal tract because AJ110941 needs strict anaerobic conditions for growth. In our study, a mono-association with the LPS producer *E. coli* induced neither hyperglycemia nor the accumulation of adipose tissues. In contrast, LPS derived from Gram-negative bacteria in the gastrointestinal tract was previously identified as one of the most important factors inducing the development of T2D (1, 3). Our results indicated that the presence of LPS in the intestinal tract was necessary, but not sufficient for the pathogenesis of diabetes. We speculated that AJ110941 may have assisted with the translocation of LPS into the blood from the intestinal tract. We are currently investigating the effects of AJ110941 on LPS translocation in *in vitro*/*vivo* models.

Regarding the particular gut bacterium involved in metabolic syndrome, *Methanobrevibacter smithii* and *Bacteroides thetaiotaomicron* were previously shown to enhance host energy storage in di-associated mice (20). Furthermore, Fei and Zhao reported that the strain *Enterobacter cloacae* B29, which was isolated from an obese human subject, induced obesity and insulin resistance accompanied by serum endotoxemia in mono-associated mice (9).

This is the first study to have successfully identified a specific *Lachnospiraceae* bacterium involved in metabolic disorders. Future studies are needed to elucidate the molecular mechanisms underlying the adverse effects of AJ110941 on glucose and lipid metabolism in mouse models. It remains unclear whether AJ110941 or its closely-related species inhabits the human intestinal tract. We intend to perform a preliminary epidemiological study on obese and diabetic subjects to address this question.

The 16S rRNA gene sequence of the isolated bacterium (strain AJ110941) is available in the DDBJ/EMBL/GenBank databases under the accession number AB861470.

## Supplementary Information



## Figures and Tables

**Fig. 1:**
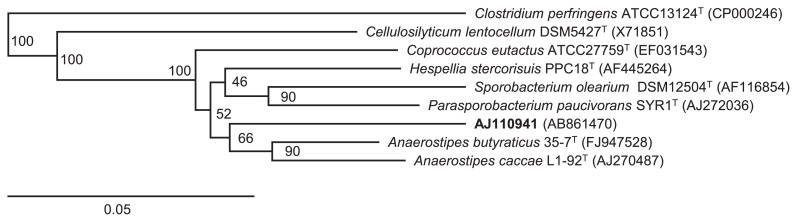
Phylogenetic tree based on the 16S rRNA gene constructed by Weighbor, the weighted neighbor-joining tree building algorithm, showing AJ110941 and other strains of the family *Lachnospiraceae*. *Clostridium perfringens* ATCC13124^T^ was used as an outgroup. The numbers close to the nodes represent bootstrap values (*n*=100 replicates). The scale bar represents 0.05 substitutions per nucleotide position.

**Table 1:** Phenotypes of germ-free and gnotobiotic *ob/ob* mice

	Group-1	Group-2	Group-3
Body weight without the caecum (g)	58.10 ± 5.98^a^	61.75 ± 4.29^a^	63.48 ± 10.37^a^
Cecum (% of body weight)	11.63 ± 4.89^a^	12.88 ± 1.94^a^	4.47 ± 0.93^b^
Liver (% of body weight without the cecum)	6.91 ± 0.66^a^	7.29 ± 1.83^a^	9.70 ± 0.46^b^
Mesenteric adipose tissue (% of body weight without the cecum)	1.64 ± 0.46^a^	1.81 ± 0.39^a^	2.63 ± 0.30^b^
Fasting blood glucose (mg dL^−1^)	216.5 ± 113.6^a^	286.5 ± 54.1^ab^	433.25 ± 65.4^b^
Fasting plasma insulin (μU mL^−1^)	339.7 ± 46.44^a^	299.8 ± 105.0^a^	159.4 ± 50.9^b^
Fasting plasma glucagon (pg mL^−1^)	179.9 ± 43.0^a^	324.2 ± 42.4^a^	529.0 ± 18.3^b^
HOMA-IR	132.5 ± 23.3^a^	187.5 ± 70.6^a^	184.4 ± 64.1^a^
HOMA-β	1350.4 ± 468.9^a^	638.5 ± 411.9^ab^	141.7 ± 40.7^b^

Group-1: germ-free, Group-2: colonization by *E. coli*, Group-3: colonization by AJ110941 with *E. coli*. Data are expressed as means±SD. Values not sharing a common letter are significantly different at *p*<0.05 by the Tukey–Kramer multiple comparisons test (*n*=4 per group) using the JMP 10.0.0 statistical software package (SAS Institute, Cary, NC).
