# Supplementary material for: Intestinal Colonization by a Lachnospiraceae Bacterium Contributes to the Development of Diabetes in Obese Mice
Source: Microbes Environ. 2014 Oct 4;29(4):427–30. doi: 10.1264/jsme2.ME14054 (PMC4262368; doi:10.1264/jsme2.ME14054)

Fig. S1. FBG levels in 5- and 11-week-old db/+ or db/db mice. FBG levels were measured after fasting for 16 h. Data are expressed as means ± SD. Differences between groups were examined for significance with the Student's t-test (n=10 per group) using the JMP 10.0.0 statistical software package (SAS Institute, Cary, NC).

Fig. S2. Comparison of gut microbiota by T-RFLP analysis between db/+ and db/db mice. Data are expressed as means±SD. The 282 bp of all fragments was the only fragment that significantly differed between db/+ and db/db mice at both 5 and 11 weeks old. Differences between groups were examined for significance with the Student's t-test (n=10 per group) using the JMP 10.0.0 statistical software package.

Fig. S3. Temporal changes in FBG levels in ob/ob mice. FBG levels were determined weekly. FBG levels were measured after fasting for 16 h. Mouse Number 4 had the highest FBG level among all ob/ob mice (purple bold line). In contrast, Mouse Number 6 had normal FBG levels and the highest body weight among all ob/ob mice (orange bold line).

Fig. S4. Comparison of gut microbiota by T-RFLP analysis between abnormal and normal glycemic ob/ob mice. The gut microbiota patterns at 5 and 11 weeks in hyperglycemic Mouse Number 4 (A) and normal glycemic Mouse Number 6 (B) as a representative example of normal glycemic ob/ob mice. The 282 bp fragment was the most dominant fragment detected in the fecal samples from Mouse Number 4.

Supplement 1, Keishi Kameyama

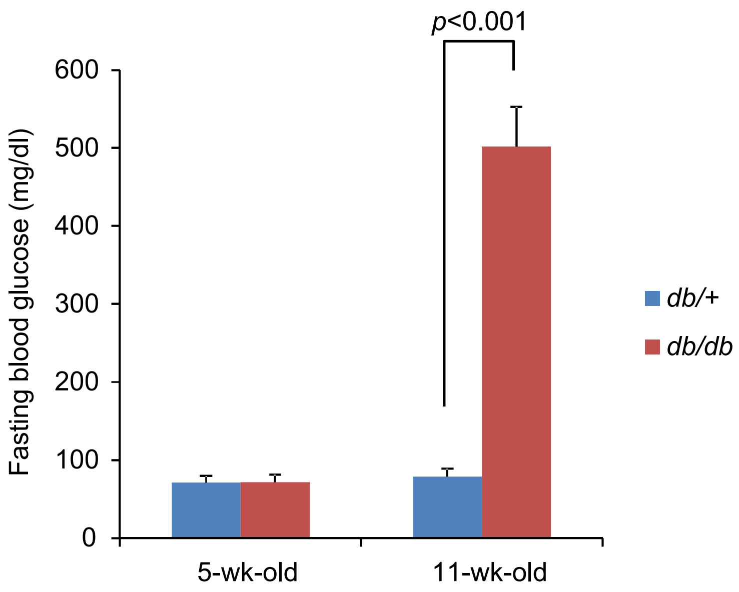

Supplement 2, Keishi Kameyama

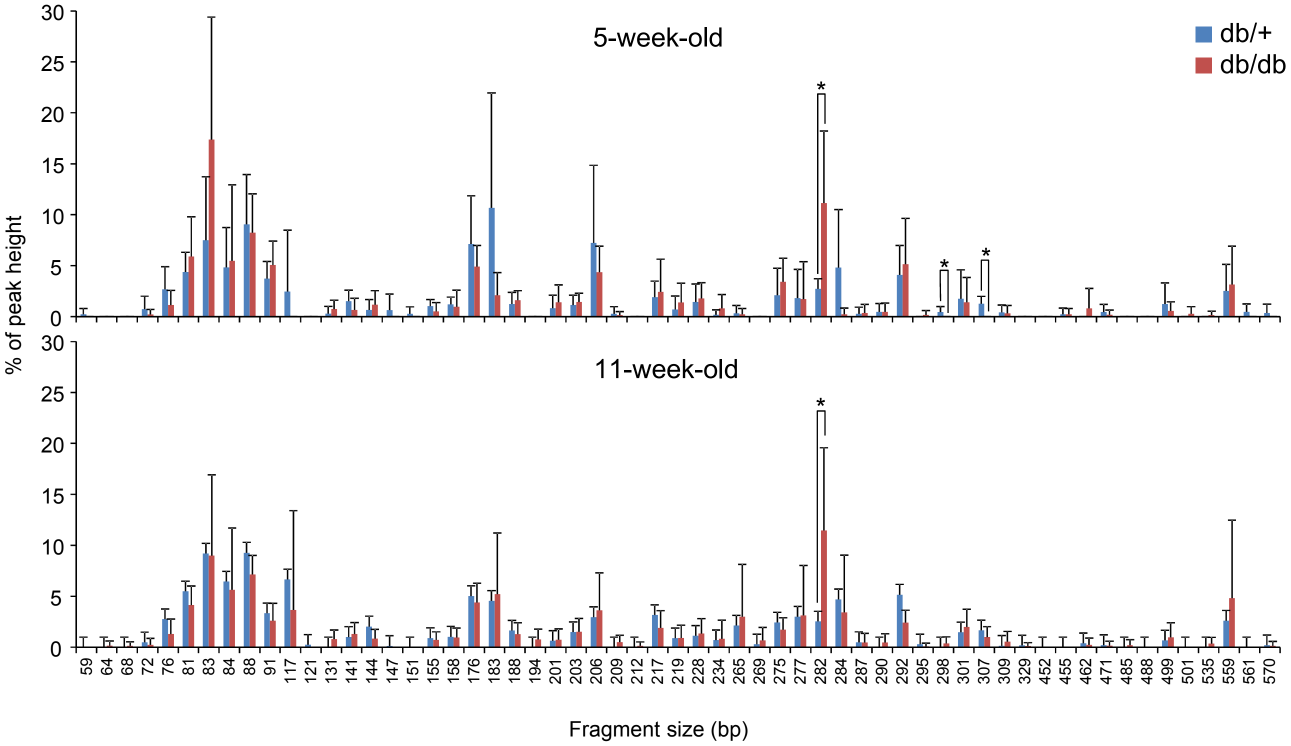

Supplement 3, Keishi Kameyama

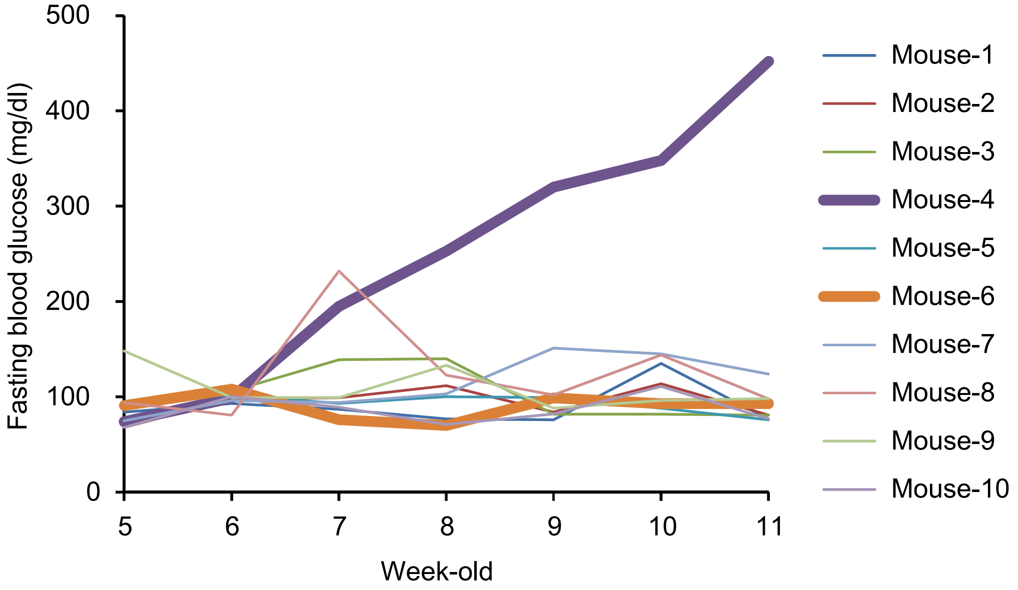

Supplement 4, Keishi Kameyama

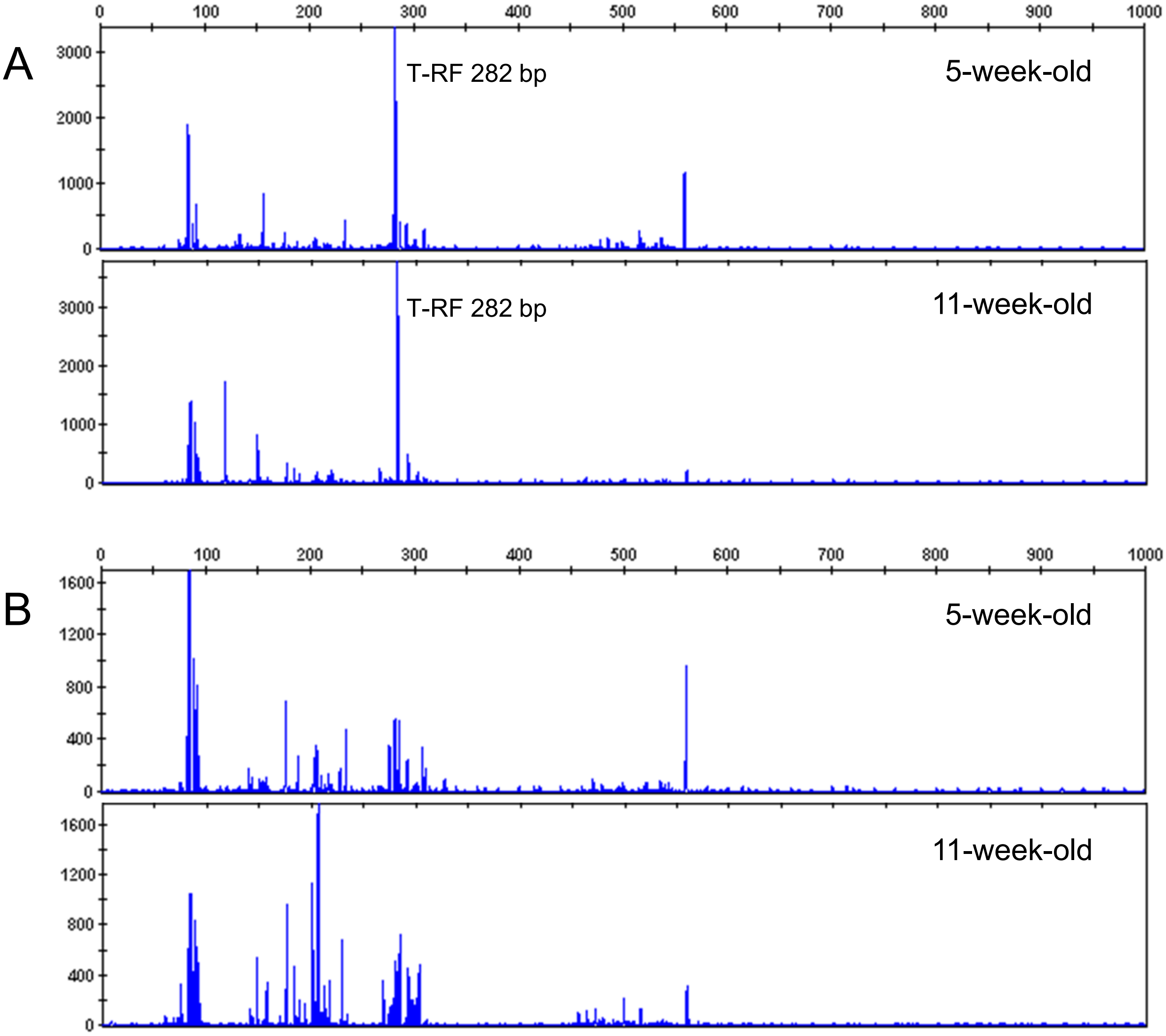

Supplement: Supplementary file 1 [file 29_427_s1.pdf]
